# Job vacancy at the European Centre for Disease Prevention and Control

**DOI:** 10.2807/1560-7917.ES.2017.22.16.30516

**Published:** 2017-04-20

**Authors:** Eurosurveillance editorial team

**Affiliations:** 1 European Centre for Disease Prevention and Control (ECDC), Stockholm, Sweden

The European Centre for Disease Prevention and Control (ECDC) invites applications for the following position:

Expert Mathematical Modelling

The deadline for application is 19 May. For more information, visit the ECDC website: http://ecdc.europa.eu/en/aboutus/jobs/Pages/JobOpportunities.aspx

